# Curli of Uropathogenic *Escherichia coli* Enhance Urinary Tract Colonization as a Fitness Factor

**DOI:** 10.3389/fmicb.2019.02063

**Published:** 2019-09-03

**Authors:** Víctor M. Luna-Pineda, Leticia Moreno-Fierros, Vicenta Cázares-Domínguez, Damaris Ilhuicatzi-Alvarado, Sara A. Ochoa, Ariadnna Cruz-Córdova, Pedro Valencia-Mayoral, Alejandra Rodríguez-Leviz, Juan Xicohtencatl-Cortes

**Affiliations:** ^1^Laboratorio de Investigación en Bacteriología Intestinal, Hospital Infantil de México “Federico Gómez”, Mexico City, Mexico; ^2^Laboratorio de Inmunidad en Mucosas, Unidad de Biomedicina, Facultad de Estudios Superiores Iztacala, Universidad Nacional Autónoma de México, Tlalnepantla, Mexico; ^3^Departamento de Patología, Hospital Infantil de México “Federico Gómez”, Mexico City, Mexico

**Keywords:** uropathogenic *Escherichia coli*, curli, urinary tract infection, fitness factor, CsgA protein

## Abstract

Curli, a type of fimbriae widely distributed in uropathogenic *Escherichia coli* (UPEC), are involved in adhesion to human bladder cell surfaces and biofilm development. The role of UPEC curli was evaluated in a murine model of urinary tract infection. The aim of this study was to establish the role of curli in C57BL/6 mice transurethrally infected with curli-producing and non-curli-producing UPEC strains. We confirmed that curli enhanced UPEC colonization in the urinary tract, resulting in damage to both the bladder and kidney. Intranasal immunization with recombinant CsgA protein protected against colonization by curli-producing UPEC in the urinary tract. Quantification of cytokines from urinary tract organs showed increases in interleukin-6 and tumor necrosis factor (TNF) release in the kidneys 48 h postinfection with curli-producing UPEC. By contrast, mice infected with non-curli-producing UPEC showed the highest release of interleukin-6, -10, and -17A and TNF. Curli may obscure other fimbriae and LPS, preventing interactions with Toll-like receptors. When intranasal immunization with recombinant FimH and PapG proteins and subsequent infection with this strain were performed, cytokine quantification showed a decrease in the stimulation and release by the uroepithelium. Thus, curli are amyloid-like fimbriae that enhances colonization in the urinary tract and a possible fitness factor.

## Introduction

Curli fimbriae are classified as amyloid fibers and are involved in surface adhesion, cell aggregation, and biofilm formation in *Escherichia coli* and other *Enterobacteriaceae* (Kikuchi et al., 2005). Curli facilitate bacterial binding with the extracellular matrix and various serum proteins, such as fibronectin, laminin, plasminogen, and plasminogen activator protein (Barnhart and Chapman, 2006). Subunits of curli and their assembly machinery are encoded on two divergently transcribed operons; the *csgBAC* operon encodes the major curli subunit CsgA, which is responsible for interaction with the host components (Hufnagel et al., 2015).

Urinary tract infections (UTIs) are one of the most important human infections and are mainly caused by uropathogenic *E. coli* (UPEC) (Flores-Mireles et al., 2015). In Mexico, UPEC-associated UTIs are the second leading cause of morbidity, with more than four million cases annually (Luna-Pineda et al., 2018a). Furthermore, UPEC clinical isolates have been shown to promote curli expression and biofilm formation at 26°C and in low nutritional conditions (Lim et al., 2014). In addition, curli expression at 37°C has been described in urinary *E. coli* isolated from patients with bacteremia (Hung et al., 2014). Curli expression was found in 74 and 89% of UPEC isolates from women with cystitis and upper urinary tract symptoms, respectively (Norinder et al., 2012), and these fimbriae were expressed in 56–60% of UPEC isolates from children with cystitis and pyelonephritis (Kudinha et al., 2013). Recently, we described a high frequency (≥95%) of the *csgA* gene in multidrug-resistant and extensively drug-resistant UPEC clinical strains obtained from pediatric patients with uncomplicated and complicated UTIs (Ochoa et al., 2016; Luna-Pineda et al., 2018b).

Curli-producing UPEC can induce interleukin (IL)-8 release in renal epithelial cells and can interact with antimicrobial peptides LL-37 without affecting bacterial colonization in the kidney (Kai-Larsen et al., 2010). Dimeric and trimeric recombinant proteins generated by our workgroup that included CsgA promoted IL-6 and IL-8 release from the supernatant of bladder HTB-5 cells. The antibodies generated against these recombinant proteins protected against UPEC adherence to HTB-5 cells (Luna-Pineda et al., 2016). The FN075 molecule is an inhibitor of CsgA-formed amyloid fiber and has the ability to attenuate UPEC virulence in a murine model of UTI (Cegelski et al., 2009). The curli fimbriae are associated with biofilm-like structures produced by UPEC and are related to the persistence of this bacterium in the urinary tract (Anderson et al., 2003; Trautner and Darouiche, 2004). Interestingly, curli are associated with adhesion *in vitro*, which is enhanced by phosphoethanolamine cellulose (Cordeiro et al., 2016; Hollenbeck et al., 2018). Based on this finding, the curli of UPEC could contribute to colonization by attach to cell surface and modifying cytokine release *in vivo*. This study evaluated the role of curli in a murine model of UTI using curli-producing and non-curli-producing UPEC strains.

## Materials and Methods

### Mutation and Phenotypic Complementation of Uropathogenic *Escherichia coli*

The UPEC clinical strain 529U-0712 was evaluated by our workgroup, beginning with 500 isolates from pediatric patients with UTI (Ochoa et al., 2016). The inclusion criteria were (1) amplification of the *csgA* gene, (2) curli expression, (3) cellulose expression, and (4) antibiotic sensitivity (gentamicin, ampicillin, kanamycin, and chloramphenicol). The *csgA* gene was inactivated using the Datsenko and Wanner method (Datsenko and Wanner, 2000). Briefly, the inactivation cassette was amplified using the pKD3 vector and specific primers (mCsgA_For 5′-GTTTTACATGAAACTTTTAAAAGTAG CAGCAATTGCAGCAATCGTATTCGTGTAGGCTGGAGCTGCTTC-3′ and mCsgA_Rev 5′-GCGCCCTGTTTCTTTCATACTGATGA TGTATTAGTACTGATGAGCGGTCGCATATGAATATCCTCCTTAG-3′), which contained the gene encoding chloramphenicol resistance. The putative transformative strains were verified by polymerase chain reaction (PCR) using the following specific primers: vCsgA_For 5′-GCCAGTATTTCGCAA GGTGC-3′ and vCsgA_Rev 5′-GGTGTACATATCCCCTTGCTGG-3′. An amplicon of approximately 1,400 base pairs (bp) and the absence of Congo red dye fixation were considered positive indicators of mutation. Mutants were confirmed by transmission electron microscopy (TEM), and phenotype complementation was generated using the pCsgGC vector. The pJcsgGC vector contained the sequences of the csgGFED and csgBAC operons cloned into the plasmid pJET1.2 blunt (Thermo Fisher Scientific, MA, USA) using the following specific primers: CsgG_For 5′-GCGAGCTCGGTTGATATTTGGTTACGC-3′ and CsgC_Rev 5′-CTCTCTTATGCTCGGCAGTTGAGCTC-3′.

### Adhesion and Invasion Assays of a Bladder and Renal Cell Lines

The human bladder cell line HTB-5 and human renal cell line HTB-47 acquired from the American Type Culture Collection (ATCC, VA, USA) was grown in Eagle’s minimum essential medium (EMEM) from ATCC. Twenty-four-well plates were prepared with 1 × 10^5^ cells/well in 1 ml of EMEM supplemented with 10% fetal bovine serum (Gibco, Thermo Fisher Scientific, MA, USA) until the cells reached 80% confluence. Previously, the UPEC strain was cultured in conditions to promote curli expression and adjusted to an OD_600_ of 1. Cells were treated with 2.5% mannose (Sigma-Aldrich Corp, MO, USA) and incubated for 1 h at 37°C under agitation. Cell monolayers were infected with 1 × 10^7^ bacteria (MOI of 100) and incubated for 3 h at 37°C with 5% CO_2_. The number of colony forming units (CFU) was determined by three methods: (1) after infection, the monolayers were treated with 20 μl of 0.5% Triton X-100 in phosphate buffer solution (PBS) and collected for quantification of total bacteria (not adhered, adhered and intracellular); (2) the infected monolayers were washed three times with 1 ml of PBS, followed by treatment with 1 ml of PBS with 0.1% Triton X-100 and collected for the quantification of adhered and intracellular bacteria; and (3) the infected monolayers were washed three times with 1 ml of PBS, incubated with 1 ml of EMEM with 100 μg/ml of gentamicin for 2 h to remove the adhering bacteria and washed three times with PBS. Finally, the treated monolayers were collected with 1 ml of PBS with 0.1% Triton X-100 for quantification of the intracellular bacteria. Quantification of CFU/mL was performed by the microdilution method described by Hannan and Hunstad (Hannan and Hunstad, 2016). EMEM was used as a negative control in these assays, and the wild-type strain was considered to show 100% adherence-invasion.

### Murine Model of Urinary Tract Infection

C57BL/6 female mice at 10–12 weeks were obtained from the “Facultad de Estudios Superiores-Iztacala” of UNAM. The UPEC strain was cultured as described above, and groups of eight mice were inoculated transurethrally with 1 × 10^8^ bacteria in 100 μl of PBS, according to the modified protocol of Hung et al. (2009). Forty-eight hours after infection, the mice were sacrificed to obtain the bladder and the kidneys, which were mechanically homogenized in 1 ml of PBS with 0.05% Triton X-100 (Sigma-Aldrich Corp., Mo, USA). Quantification of CFU/organ was performed as described above. PBS was used as a negative control in this assay, and the wild-type strain was considered to show 100% colonization.

### Histology and Microscopy

Three C57BL/6 mice per group were infected and sacrificed as described above. Bladders and kidneys were obtained and fixed with 10% paraformaldehyde for hematoxylin-eosin staining. Tissue samples were embedded in paraffin, stained with hematoxylin, and sectioned by the “Departamento de Patología” of “Hospital Infantil de México Federico Gómez” (CDMX, México).

The bladder lumen is delimited by a stratified transitional epithelium of three or four cell layers, commonly termed the urothelium, and the epithelial cells are organized into the basement membrane and the lamina propria. The bladder urothelium comprises basal and intermediate epithelial cells underlying a layer of binucleate superficial facet cells (Hung et al., 2009). Cystitis scores were determined as follows: 0 = no significant lesions, 1 = few and occasional PMN cells in the stroma or lumen, as well as occasional perivascular lymphoid infiltration; 2 = presence of PMN cells and moderate edema; 3 = many PMN cells and severe edema. The kidneys basically consist of a capsule formed by connective tissue, a cortex including the renal corpuscles of the nephrons, the medulla above a striated zone (renal pyramid), and a collecting structure (calyx) near the renal pelvis and ureters (Cardiff et al., 2014). Pyelonephritis scores were determined as follows: 0 = no significant lesions; 1 = few and occasional PMN cells in the renal pelvis; 2 = rafts and/or scattered focal aggregates of PMN cells in the renal pelvis, as well as peripelvic inflammation; 3 = presence of numerous and large focal PMN cells in all tissue sections and inflammation in the parenchyma.

### Curli-Induced Cytokine Release in the Urinary Tract

Five C57BL/6 mice per group were infected and sacrificed as described above. The bladders were obtained and weighed, sectioned five times, and sonicated in 500 μl of PBS with a pulse of 10 s and 70% amplitude (ultrasonic processor; Cole-Parmer; IL; US). Both kidneys were also obtained and weighed, and they were sectioned 10 times and sonicated in 500 μl of PBS with three pulses of 10 s and 70% amplitude. In both cases, they were supplemented with cOmplete™, Mini Protease Inhibitor Cocktail (Merck KGaA, Darmstadt, Germany). The organ lysates were centrifuged for 10 min at 4,000 rpm, and the supernatants were divided into aliquots in microtubes and stored at −70°C until use. The cytokines included in this study were IL-2, IL-4, IL-6, IL-10, IL-17, IFN-γ, and tumor necrosis factor (TNF). The cytokine release was quantified using a BD Cytometric Bead Array (CBA) Mouse Th1/Th2/Th17 Cytokine Kit (Becton, Dickinson Company, BD Biosciences, CA, USA) and a BD FACSCalibur™ flow cytometer. PBS was used as a control of cytokine basal release in this assay, and the wild-type strain was considered to show 100% cytokine release in a UTI. Data analysis was executed with FCAP Array software version 3.0 of BD Biosciences and FlowJo software version 7.6 (FlowJo, LLC, OR, USA).

### Intranasal Immunization Scheme

C57BL/6 female mice at 10–12 weeks were immunized intranasally with CsgA, PapG, and FimH recombinant proteins. The first immunization was carried out with 100 μg of protein in 40 μl (20 μl in each nostril) and two boosts with 50 μg of protein in the same volume (days 7 and 14). The urine samples were recollected (days 18-20) and antibodies identified by Enzyme-Linked ImmunoSorbent Assay. On day 21, the challenge was performed as described above (murine model of UTI), using 108 bacteria in 100 μl of PBS. On day 23, the immunized and infected mice were sacrificed, and the organs were obtained for later use.

### Statistical Analysis

Student’s *t*-test was used to compare differences between means, two-way ANOVA was used to compare means among multiple groups, and the Mann-Whitney U-test was used to compare medians. The analysis and graphics were performed using GraphPad Prism software (version 7). A *p* value <0.05 was considered statistically significant for all results.

### Ethics Statement

The study was reviewed and approved by the Research Committee (Dr. Juan Garduño Espinosa), Ethics Committee (Dr. Luis Jasso Gutierrez), and Biosecurity Committee (Dr. Marcela Salazar Garcia) of “Hospital Infantil de México Federico Gómez” (HIMFG), with permit numbers HIM/2016/099 SSA.1329, HIM/2017/002 SSA.1298, HIM/2017/136 SSA.1456, and HIM 2017-107FF SSA.1421. The UPEC clinical strains were isolated from pediatric patients with UTIs and were obtained from a pre-existing collection (Ochoa et al., 2016). The aforementioned committees provided approval to use the samples from the pre-existing collection, and all samples were anonymized. The physicians from the Infectology Department asked the patients for their permission and consent to use the collected samples employed in this study. Animal assays were performed according to the specifications of the Mexican official standard NOM-62-ZOO-199, which includes technical specifications for the production, care and use of laboratory animals.^1^ Animal assays included a murine model of UTI, mouse IN immunizations, histology sections, and cytokine quantification from mouse bladders and kidneys.

### Data Availability

The datasets generated and/or analyzed during the current study are available from the corresponding author on reasonable request. Additionally, all datasets are available in the repository *via* the following web link: https://github.com/vlunapineda/Frontiers-in-Cell-and-Infect-Microb.

## Results

The UPEC clinical strain 529U-0712 (wild type) was selected based on the presence of the *csgA* gene, Congo red fixing, antibiotic resistance (ampicillin, chloramphenicol, and kanamycin), and curli expression at 37°C (Supplementary Table S1). This strain was used to generate an isogenic mutant in the *csgA* gene (*csgA*::Cm), which was restored through complementation with the plasmid pCsgG-C (complemented strain). The genotypic verification of the mutation was carried out by PCR. Amplicons of 788 bp for the wild-type strain, 1,400 bp for the mutant strain, and 788 bp for the complemented strain were obtained (Supplementary Figure S1). A wild-type strain grown on yeast extract and casamino acid (YESCA) agar supplemented with Congo red showed a high level of fixation of this dye; however, loss of retention was observed in the mutant strain, and fixation restoration was observed in the complemented strain (Supplementary Figure S1). Curli are considered an amyloid-like structure and are characterized as thin fimbria that form bunches similar to curls. The negative-staining micrographs by TEM showed these fine structures in the wild-type and complemented strains, while a loss of these structures was observed in the mutant strain (Supplementary Figure S1).

**Figure 1:**
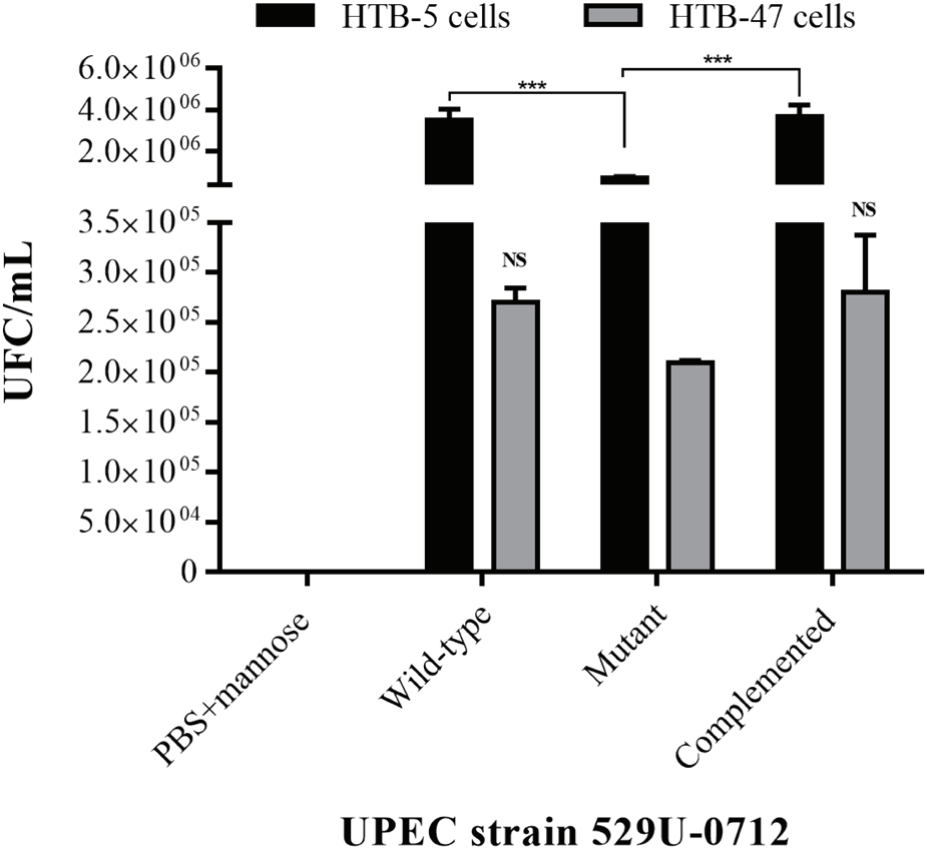
Assays of adherence to bladder HTB-5 cells and renal HTB-47 cells. The wild-type and complemented strains showed similar adhesion profiles, and a significant reduction (*p* < 0.001) in the mutant strain was observed in bladder cells. In renal cells, no significant differences were identified among all UPEC strains. Quantification of CFU/ml was performed by a plaque microdilution method. The bars represent the mean ± S. E. of three independent experiments. Uninfected cells were used as a control in this experiment. NS = not significant (*p* > 0.5); *** < 0.001.

To confirm the role of curli in adherence, we performed adhesion and invasion assays on human bladder (HTB-5) and renal (HTB-47) cells. The quantification of CFU showed an adhesion mean of 3.5 × 10^6^ CFU/ml for the wild-type strain and a decreased adhesion mean of 7.3 × 10^5^ CFU/ml for the mutant strain to bladder cells. According to the statistical analysis, the decrease in adherence produced a significant change of 79.1% (*p* < 0.0001), including the complemented strain with *p* < 0.0001 compared with the mutant strain (Figure 1). The quantitative analysis of adhesion to renal cells showed an adhesion mean of 2.7 × 10^5^ CFU/ml for the wild-type strain and an adhesion mean of 2.1 × 10^5^ CFU/ml for the mutant strain; this difference was not statistically significant, nor was the statistical difference between either and the adhesion mean of the complement strain (Figure 1). Additionally, the testing of bacterial invasion of the bladder HTB-5 and HTB-47 cells using mannose (blocking type 1 fimbriae) and gentamicin treatment (removing attached bacteria) did not show the growth of intracellular bacteria (data not shown).

### Curli-Producing Uropathogenic *Escherichia coli* Efficiently Colonizes the Mouse Urinary Tract

**Figure 2:**
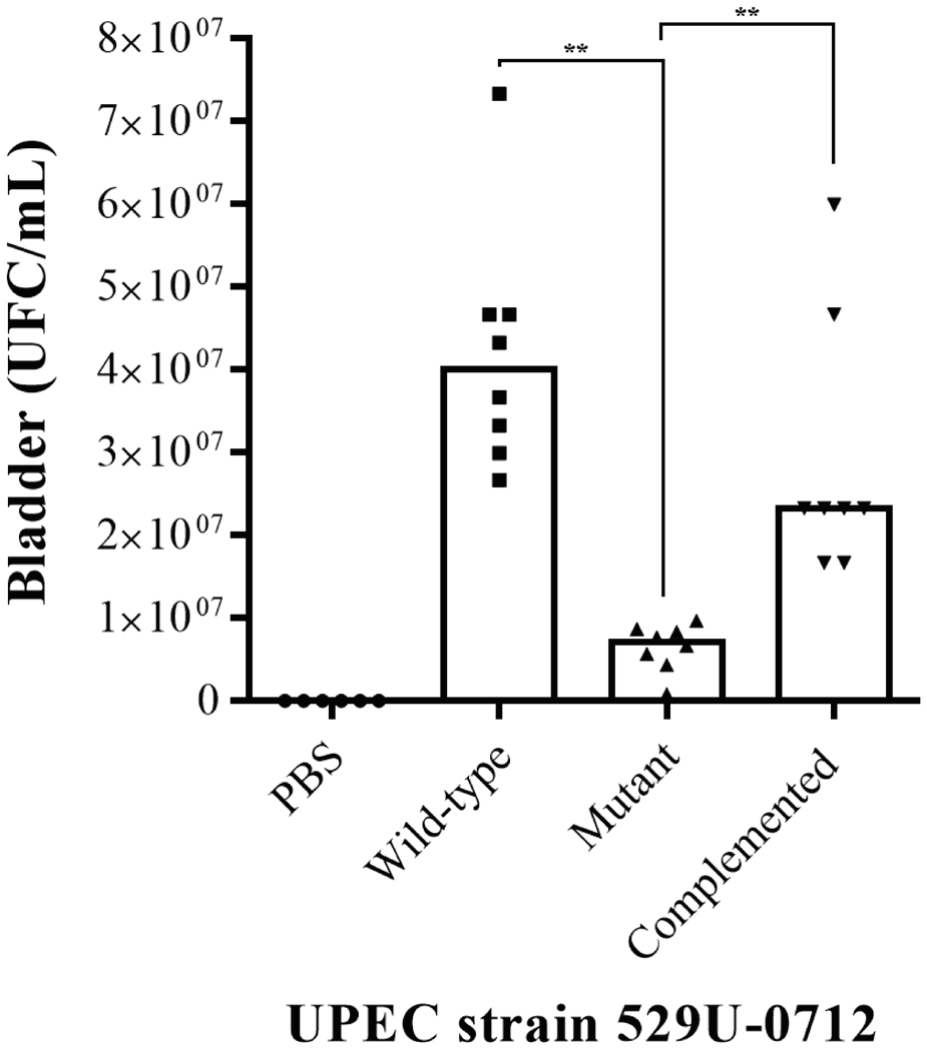
Evaluation of adherence in UPEC strain-infected mouse bladders. The murine model of UTI was generated in female C57BL/6 mice at 10–12 weeks with a transurethral inoculation of 1 × 10^8^ bacteria in 100 μl. Forty-eight hours postinfection, the mice were sacrificed to obtain the bladders, which were homogenized in PBS, and bacteria per organ (CFU/ml) were quantified by a plaque microdilution method. The UPEC clinical strain 529U-0712 was defined as the wild-type strain, the UPEC strain 529U-0712 *csgA*::Cm was defined as the mutant strain, and complementation with the plasmid pJcsgG-C resulted in the complemented strain. Transurethral inoculation with PBS was used as a control in this experiment. ** = 0.01-0.001.

**Figure 3:**
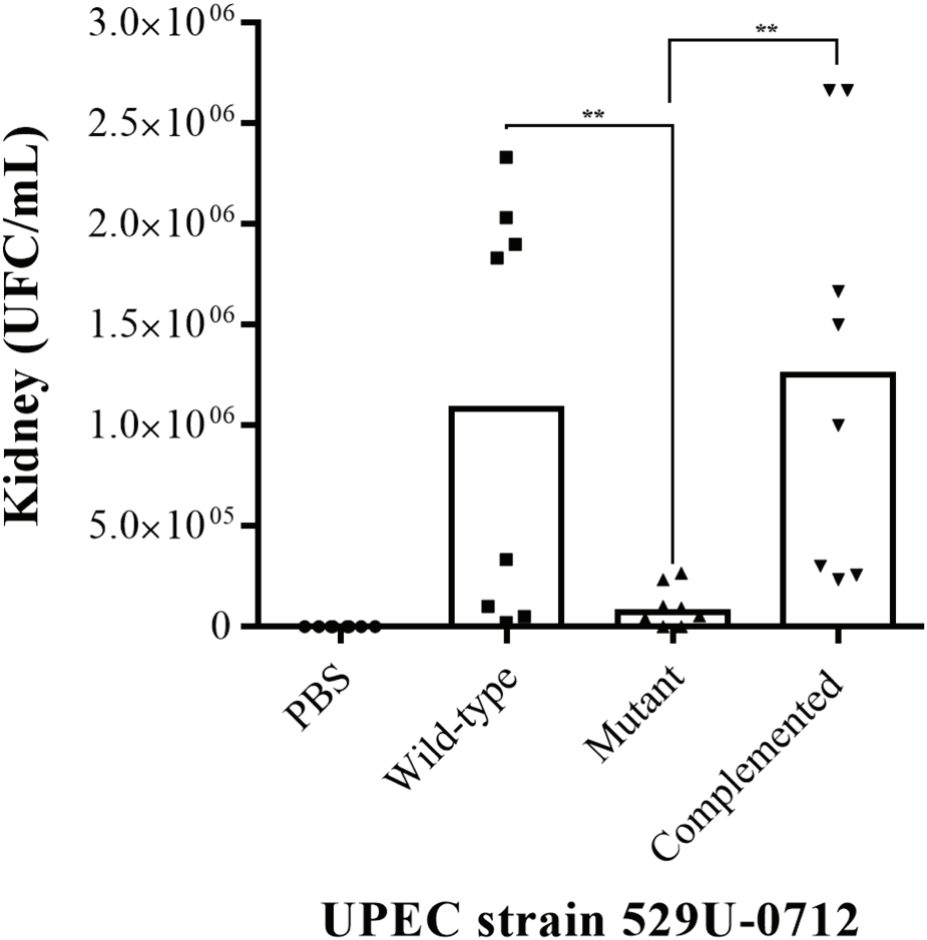
Evaluation of adherence in UPEC strain-infected mouse kidneys. The murine model of UTI was generated in female C57BL/6 mice at 10–12 weeks with a transurethral inoculation of 1 × 10^8^ bacteria in 100 μl. Forty-eight hours postinfection, the mice were sacrificed to obtain the kidneys, which were homogenized in PBS, and bacteria per organ (CFU/ml) were quantified by the plaque microdilution method. The UPEC clinical strain 529U-0712 was defined as the wild-type strain, the UPEC strain 529U-0712 *csgA*::Cm was defined as the mutant strain, and complementation with the plasmid pJcsgG-C resulted in the complemented strain. Transurethral inoculation with PBS was used as a control in this experiment. ** = 0.01-0.001.

The role of curli UPEC in the urinary tract was evaluated using a murine model of UTI in C57BL/6 female mice at 10–12 weeks of age. Bacterial quantification in the infected mouse bladder showed a significant decrease (*p* = 0.0078), with a median of 6.66 × 10^6^ CFU/ml for the mutant strain compared to a median of 4 × 10^7^ CFU/ml for the wild-type strain and a median of 2.33 × 10^7^ CFU/ml for the complemented strain (*p* = 0.0012; Figure 2). Under the same conditions, the bacterial quantification in infected mouse kidneys showed a significant reduction (*p* = 0.0078), with a median of 7.33 × 10^4^ CFU/ml for the mutant strain compared to a median of 1.08 × 10^6^ CFU/ml for the wild-type strain and a median of 1.25 × 10^6^ CFU/ml for the complemented strain (*p* = 0.0054; Figure 3).

**Figure 4:**
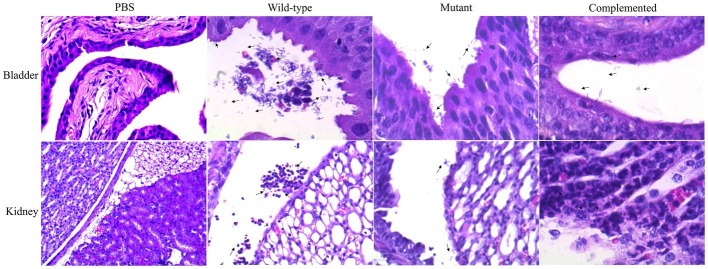
Visualization of UPEC in the bladder and kidney sections. Tissue samples were embedded in paraffin, sectioned, stained with hematoxylin-eosin, and visualized. Black arrows show the presence of bacteria as follows: PBS (control), wild-type strain (UPEC strain 529U-0712), mutant strain (*csgA*::Cm), and complemented strain (plasmid pJcsgG-C).

Histological sections of mouse bladder and kidneys were stained with hematoxylin-eosin and visualized under light microscopy. In the bladder, the adhered wild-type strain was visualized on the surface of the uroepithelium, and invading bacteria were also observed 48 h postinfection (Figure 4). Additionally, the mutant strain was localized in the bladder lumen, with low-adherence and low-invasion bacteria on the uroepithelium (Figure 4). In the kidney, the UPEC wild-type strain was visualized in the renal pelvis, and low bacteria levels were observed in the renal tubules, while the mutant strain was slightly visible in the renal pelvis and barely detectable in the tubules (Figure 4). Curli expression *in vivo* was determined by Western blotting (WB) using sonicated tissues at 48 h postinfection with collected UPEC strains and anti-CsgA antibodies (Supplementary Figure S2).

### The CsgA Structural Protein of Curli Protects Against Colonization of Uropathogenic *Escherichia coli* in Immunized Mice

**Figure 5:**
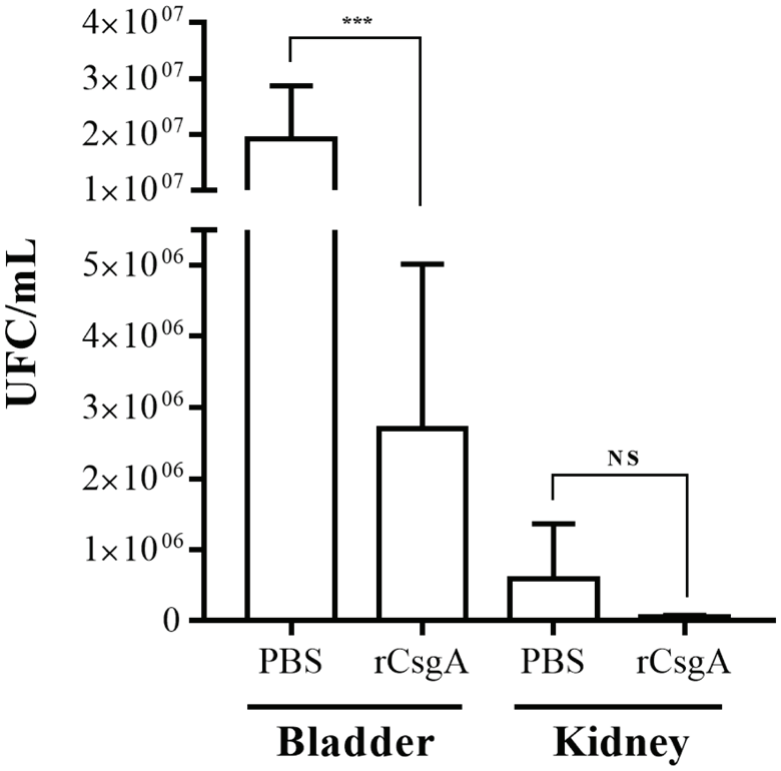
Recombinant CsgA protein immunization generates protection against colonization of UPEC. C57BL/6 female mice at 10–12 weeks were immunized intranasally. The first immunization was carried out with 100 μg of rCsgA protein in 40 μl and two boosts with 50 μg of protein in the same volume. The challenge was performed, and CFU/ml quantification was performed in both infected mouse bladders and kidneys. NS = not significant (*p* > 0.5); *** < 0.001.

Recombinant CsgA (rCsgA) protein was obtained in a previous study (Luna-Pineda et al., 2016). C57BL/6 female mice were immunized intranasally with rCsgA protein and later infected transurethrally with the UPEC strain 529U-0712. Bacterial quantification of UPEC in the total bladder showed a significant reduction (*p* = 0.0003), with an arithmetical median of 2.7 × 10^6^ CFU/ml for rCsgA protein and 1.9 × 10^7^ CFU/ml for PBS immunization (Figure 5). Nevertheless, the quantification in both kidneys showed arithmetical medians of 5.9 × 10^5^ UFC/ml for PBS immunization and 5.2 × 10^4^ UFC/ml for rCsgA immunization with no significant difference (Figure 5). Interestingly, anti-rCsgA antibodies were identified from the urine following IN immunization with rCsgA to C57BL/6 mice (Supplementary Figure S3). These data showed protection against UPEC and confirmed that curli played a role in bacterial colonization in the urinary tract.

### Curli-Producing Uropathogenic *Escherichia coli* Generate Damage and an Inflammatory Process in the Mouse Urinary Tract

**Figure 6:**
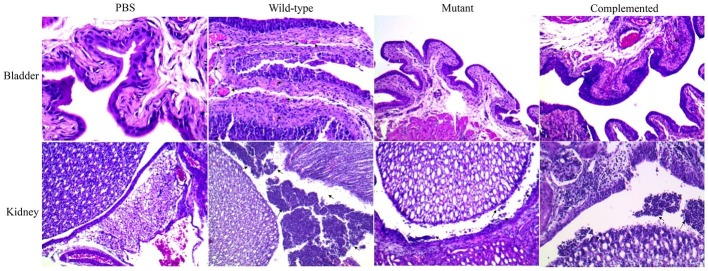
Visualization of damage in the bladder and kidney sections. Tissue samples were paraffin-embedded, sectioned, hematoxylin-eosin-stained, and visualized. Cystitis scores were determined as follows: 0 = no significant lesions; 1 = few and occasional polymorphonuclear (PMN) infiltration in the stroma or lumen, as well as few and occasional perivascular lymphoid microinfiltration; 2 = presence of PMN cells and moderate edema; 3 = many PMN cells and severe edema. Pyelonephritis scores were determined as follows: 0 = no significant lesions; 1 = few and occasional PMN cells in the renal pelvis; 2 = rafts and/or scattered focal aggregates of PMN cells in the renal pelvis, as well as peripelvic inflammation; 3 = presence of numerous and large focal PMN cells in all tissue sections and inflammation extending into the parenchyma.

Histological sections of mouse bladder after infection with wild-type UPEC strains visualized by light microscopy showed cystitis with a score of 3. The micrographs showed the presence of polymorphonuclear (PMN) infiltration in the perivascular submucosa and intraepithelial regions and a PMN cumulus in the lumen bladder with strong edema and cellular exfoliation (Figure 6). Cystitis (score = 1) in the mutant group was less severe than that in the wild-type group and showed moderate perivascular PMN infiltration (Figure 6). In the mouse kidney, pyelonephritis with a score of 3 was visualized for the UPEC wild-type strain, and numerous, large focal PMN cells were observed in all tissue sections (intra- and interepithelial sections). Furthermore, PMN cumuli in the pelvis lumen and moderate inflammation of the renal parenchyma were observed (Figure 6). A low pyelonephritis score of 1 was observed in the mutant group, which was characterized by mild inflammation of the renal parenchyma and intraepithelial PMN detection without infiltration into the renal pelvis (Figure 6).

### Curli-Producing Uropathogenic *Escherichia coli* Modify Cytokine Release in the Mouse Urinary Tract

**Figure 7:**
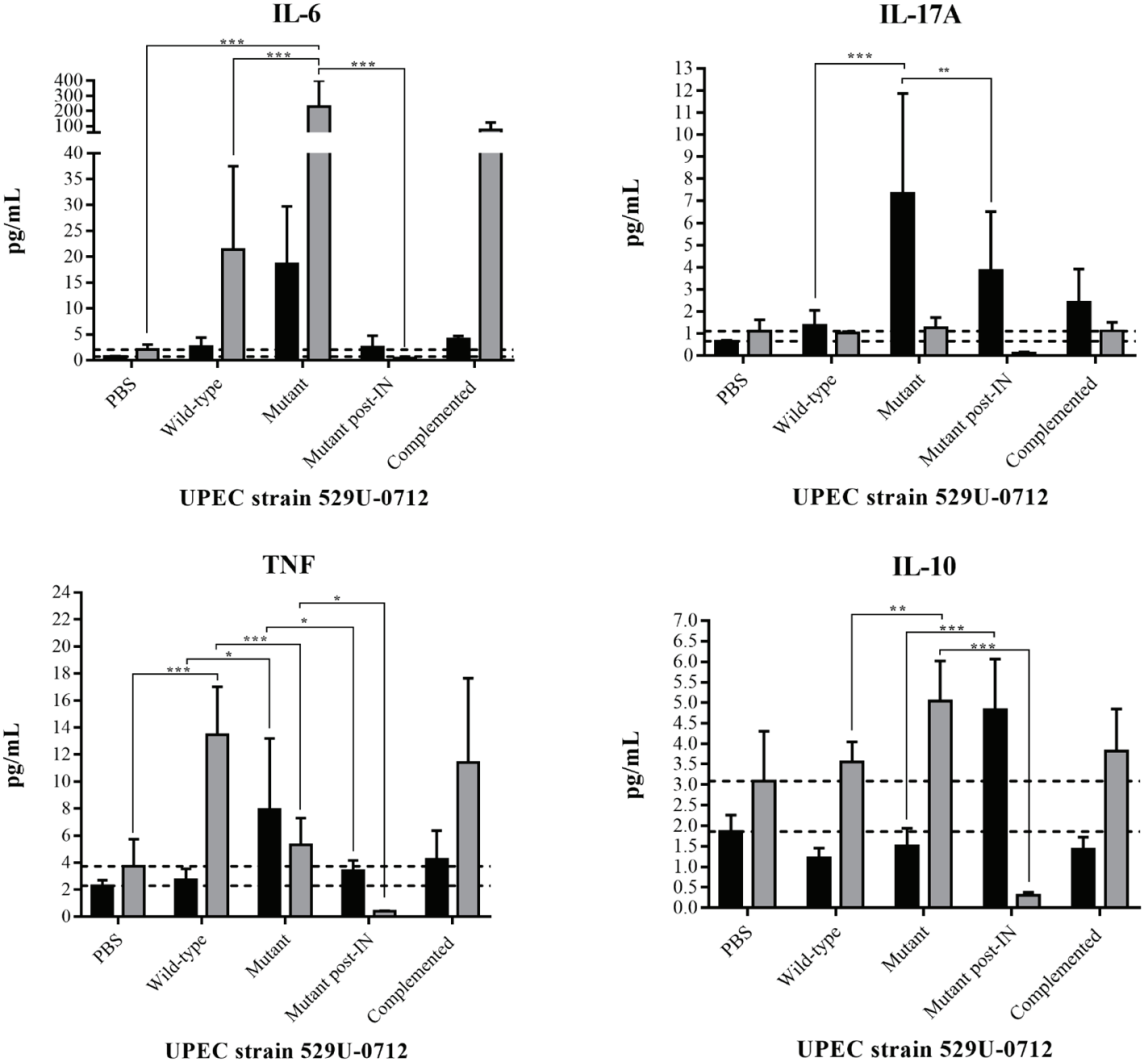
Quantification of cytokine release in the mouse bladder and kidneys. Bladders (black bars) and kidneys (gray bars) were obtained, weighed, sectioned, and sonicated. The lysate organs were centrifuged, and the supernatants were used to quantify the cytokines. The UPEC clinical strain 529U-0712 was defined as the wild-type strain, the UPEC strain 529U-0712 *csgA*::Cm was defined as the mutant strain, and its complementation with the plasmid pJcsgG-C resulted in the complemented strain. Additionally, IN immunization followed by infection with the mutant strain was included and defined as mutant post-IN. Transurethral inoculation with PBS was used as a control in this experiment. * = 0.05-0.01; ** = 0.01-0.001; *** < 0.001.

Cytokine release in the bladder and kidneys of infected mice was quantified to confirm an inflammatory response (Figure 7; Supplementary Figure S4). In wild-type strain-infected mice, IL-6 release in the kidney was significantly increased (*p* = 0.0001), with a release of 21.4 pg/ml, compared to that of PBS-infected mice; however, a nonsignificant (*p* = 0.9573) increase was observed in the bladder. In mutant strain-infected mice, an IL-6 release of 227.2 pg/ml was observed in the kidneys, representing a significant increase (*p* < 0.0001) compared to that in the PBS-infected mice; however, no significant difference in release (*p* = 0.6618) was observed in the bladder (Figure 7; Supplementary Table S2). We hypothesized that the absence of curli results in the exposure of other structures, such as FimH of type 1 fimbria and PapG of P fimbria. Interestingly, an intranasal (IN) immunization scheme with these recombinant proteins following infection with the mutant strain resulted in a significant decrease (*p* < 0.0001) in this cytokine (Figure 7; Supplementary Table S2).

Similarly, IL-17 release in the bladder showed a significant increase of 7.3 pg/ml (*p* < 0.0001), but no significant difference was observed between PBS- and wild-type strain-infected mice. Under the same conditions and using the mutant strain, a decreased IL-17A release of 3.8 pg/ml in the bladder (*p* = 0.0032) was observed with previous IN immunization with rFimH and rPapG, although no significant changes were observed in the kidneys (Figure 7; Supplementary Table S2).

TNF release was quantified in the kidneys and showed a value of 13.4 pg/ml in wild-type strain-infected mice with *p* < 0.0001 compared with PBS-infected mice. A significant decrease (*p* < 0.0001) in this cytokine was identified in the kidneys of mutant strain-infected mice (5.3 pg/ml) and after previous IN immunization with rFimH and rPapG (0.4 pg/ml). In the bladder, a significant increase (*p* = 0.0057) in TNF release with a value of 7.9 pg/ml was identified in mutant strain-infected mice, but a significant decrease to 3.4 pg/ml was observed post-IN immunization (Figure 7; Supplementary Table S2).

IL-10, an anti-inflammatory cytokine, was also quantified in infected mice, and similar values were obtained in both the bladder and kidneys of PBS- and wild-type strain-infected mice. However, in the kidneys but not in the bladder of mutant strain-infected mice, a significant increase (*p* = 0.0037) of 3.5–5.0 pg/ml was identified for this cytokine. Interestingly, in IN-immunized and mutant strain-infected mice, this cytokine showed a significant increase (*p* < 0.0001) of 4.8 pg/ml in the bladder and a decrease (*p* < 0.0001) of 0.3 pg/ml in the kidneys (Figure 7; Supplementary Table S2). IL-2, IL-4, and interferon (IFN)-γ cytokine release was similar in both the bladder and kidneys between groups of PBS- and wild-type strain-infected mice; however, a significant decrease (*p* < 0.0001) was observed in both organs after IN immunization, most likely due to the absence of UPEC colonization in the urinary tract (Supplementary Figure S4, Supplementary Table S2).

## Discussion

UPEC colonization in the urinary tract depends on virulence and fitness factors, such as fimbriae, iron uptake, toxins, flagella, autotransporter proteins, and the capsule (Subashchandrabose and Mobley, 2015). Curli, a type of amyloid-like fimbriae, are considered a virulence factor due to their role in adherence to bladder cells and biofilm formation (Kikuchi et al., 2005; Cordeiro et al., 2016; Hollenbeck et al., 2018). In this study, we confirmed an association of curli with adherence to human bladder cells using a mutant in the *csgA* gene from the clinical strain of UPEC (529U-0712), while the wild-type strain was isolated from the urine of a pediatric patient. These data were consistent with those of other studies performed with HTB-9 (human bladder carcinoma), Vero (African green monkey kidney cells), and HUVEC (human umbilical vein) cell lines. In this study, we included a complemented UPEC strain that confirmed the polar noneffect associated with the selection gene and its promoter (Cordeiro et al., 2016; Hollenbeck et al., 2018). Nevertheless, a role of curli in the colonization of the urinary tract *in vivo* has poorly been described (Kai-Larsen et al., 2010; Nhu et al., 2018). We established a murine model of UTI by transurethral inoculation in C57BL/6 female mice. The noncurliated UPEC strain (mutant) showed a decrease in colonization in the mouse bladder, which was confirmed by histological sections stained with hematoxylin-eosin. However, rCsgA protein-immunized mice showed protection against bladder colonization by the curliated UPEC strain (wild-type). Additionally, CsgA was shown to exist in a polymeric form in the bladder and a monomeric form in the kidneys of infected mice. Urine in the urinary tract has a variable composition; urine from the bladder is more osmolar than urine from the renal pelvis (Cahill et al., 2003). High osmolarity triggers a two-component regulatory system (EnvZ/OmpR), where phosphorylated OmpR binds to the *csgDEFG* promoter and actives *csgD* transcription (Prigent-Combaret et al., 2001). The CsgD protein is the positive regulator that activates *csgBA* transcription, generating high CsgA protein concentrations (Hammar et al., 1995). A high CsgA concentration generates polymerization, forming CsgA oligomers and fibril-like structures (Wang et al., 2007).

Histological sections (with hematoxylin-eosin staining) of the bladder and kidneys from infected mice showed damage and inflammatory processes. Histological changes after UPEC infection were observed, showing inflammatory processes in the perivascular submucosa with intra- and interepithelial PMN cells as well as the presence of adhered and intracellular bacteria. These results indicated acute cystitis when mice were infected with the wild-type strain; however, decreased in damage and in identified bacteria was observed in the bladder lumen of mice infected with the mutant strain. Acute pyelonephritis was observed in mice infected with the wild-type strain and was characterized by pelvis inflammation, inter- and intratubular PMN infiltration, and the presence of bacteria and abnormal access in the renal parenchymal. By contrast, mutant strain-infected mice showed the least severe damage and lowest levels of bacteria in the kidney. Our data confirmed that curli are a colonization factor, which has been described in bladder cell lines by other groups (Kai-Larsen et al., 2010; Cordeiro et al., 2016; Hollenbeck et al., 2018; Nhu et al., 2018). A role of curli in kidney colonization was also shown in a C57BL/6 mouse model of UTI; however, these fimbriae are not involved in adherence to kidney line cells. Curlicides have been shown to attenuate the virulence of the UPEC strain UTI89, showing a decrease in the colonization and intracellular bacterial community in a murine model of UTI (Cegelski et al., 2009). To confirm this finding, we performed IN immunization with rCsgA in a C57BL/6 mouse model, generating antibodies and protection against UPEC colonization. Previously, we showed that polyclonal antibodies against CsgA inhibited the adherence of the UPEC strain CFT073 to the bladder cell line (Luna-Pineda et al., 2016).

The epithelial cells in the urinary tract are the first line of defense and secrete soluble compounds such as proinflammatory cytokines (Abraham and Miao, 2015). Fimbrial adhesins, such as pathogen-associated molecular patterns (PAMP), are associated with cytokine release and are capable of recognizing pattern recognition receptors, including Toll-like receptor (TLR) 4, in the mucosa of the urinary tract (Sirard et al., 2006). CsgA, a structural protein of curli, interacts with the TLR2-TLR1 dimer to produce IL-6 release in marrow-derived macrophages (Rapsinski et al., 2015). Our previous research identified TLR2 and TLR4 expression in the bladder cell line and showed the release of the cytokines IL-6 and IL-8 by rCsgA protein (Luna-Pineda et al., 2016). Mice infected with curliated UPEC strains (wild-type and complemented strains) also released IL-6 in the kidney, but the highest release of this cytokine was found for the mutant strain.

Synergistically, LPS, type 1 fimbriae and P fimbriae mainly activate the release of IL-6 *in vitro* and *in vivo via* TLR4 (Frendeus et al., 2001; Hedlund et al., 2001; Samuelsson et al., 2004; Song et al., 2007). According to our data, we hypothesized that curli might hide the other fimbriae and LPS, preventing interaction with TLR4 and the release of IL-6. In addition, we identified specific IgG antibodies against FimH and PapG proteins in mouse urine following IN immunization using rFimH and rPapG (data not shown). Interestingly, IN immunization with these recombinant proteins followed by infection with the mutant strain resulted in a strong decrease in the release of IL-6 in the kidney, indicating that curli hide these fimbriae, which could be an immune response evasion strategy of UPEC. Intracellular UPEC can generate the release of IL-6 in model mice of UTI, which can be decreased by forskolin action, maintaining the extracellular localization of the bacteria (Bishop et al., 2007). This finding could explain why infection with a mutant strain after previous IN immunization (rFimH and rPapG) decreases the release of this cytokine.

IL-17A showed a similar effect in the bladder tissue, but IN immunization resulted in a small decrease in this cytokine. This result suggests that curli could synergistically function with type 1 and P fimbriae in the activation of γδ T cells. These T cells have been identified as the main cells that release IL-17A and have a role in activating the innate immune response in the mouse bladder (Sivick et al., 2010). These data explained why decreased inflammation was observed in histological sections from mouse bladders with little PMN infiltration. Nonetheless, TNF, an inflammatory cytokine, was released in the kidney but not in the bladder when mice were infected with the curliated UPEC strain. Ly6C^+^ macrophages produce the cytokine TNF in response to infection, which activates resident Ly6C^−^ macrophages to secrete CXCL2 and promote migration of neutrophils (Schiwon et al., 2014). Histological sections showed a high number of PMN cells in both the bladder and kidney. The noncurliated UPEC strain showed a decrease in TNF, while after IN immunization followed by infection with this strain, TNF was absent in the kidney, indicating synergistic activation between curli and other fimbriae (type 1 and P fimbriae). UPEC adheres to NK cells through the type 1 fimbria, activating and releasing TNF as an innate immune response (Gur et al., 2013). In the bladder, a contrary response was identified; when mice were infected with noncurliated UPEC, an increase TNF was detected, but TNF was decreased in post-IN immunization mice. These data also showed that curli might hide the type 1 fimbria, preventing interaction with bladder-resident NK cells and the release of TNF.

IL-10, a master regulator of innate immunity, was identified 2 h postinfection from uroepithelial cells and monocytes and had a protective role in both the bladder and kidney (Duell et al., 2012, 2013). Six hours postinfection, bladder mast cells were shown to release this cytokine, preventing the expression of costimulatory molecules on dendritic cells (Chan et al., 2013). Mice infected with the noncurliated UPEC strain showed the highest release of this cytokine in the kidney, probably due to curli blocking other fimbriae that activate epithelial cells or monocytes 48 h postinfection. Interestingly, IL-10 from the bladder was identified only in mice post-IN immunization that were infected with the noncurliated UPEC strain, confirming that type 1 fimbria was associated with the regulation of this cytokine, as described for the UPEC mutant strain deficient in FimH (Duell et al., 2012). Other cytokines (IL-2, IL-4, and INF-γ) did not show changes in either organ with any UPEC strain.

In conclusion, the curli of UPEC are a fitness factor that enhances colonization in the urinary tract and could be considered a strategy for evasion of the immune system; however, it is necessary to perform other experiments to confirm this hypothesis in the future.

## Data Availability

All datasets generated for this study are included in the manuscript and/or the Supplementary Files.

## Ethics Statement

The animal study was reviewed and approved by Research, Ethics and Biosecurity Committees of “Hospital Infantil de México Federico Gómez.”

## Author Contributions

VL-P and JX-C wrote the main manuscript. VL-P, VC-D, DI-A, and LM-F prepared Figures 1–3, 5. VL-P, SO, AR-L, and PV-M prepared Figures 4, 6. VL-P, AC-C, DI-A, and LM-F prepared Figure 7. VL-P, VC-D, SO, and AC-C prepared the supplementary information. VL-P and JX-C reviewed the manuscript.

### Conflict of Interest Statement

The authors declare that the research was conducted in the absence of any commercial or financial relationships that could be construed as a potential conflict of interest.

## Footnotes

^1^https://www.gob.mx/senasica/documentos/nom-062-zoo-1999
