# Supplementary material for: Curli of Uropathogenic Escherichia coli Enhance Urinary Tract Colonization as a Fitness Factor
Source: Front Microbiol. 2019 Sep 3;10:2063. doi: 10.3389/fmicb.2019.02063 (PMC6733918; doi:10.3389/fmicb.2019.02063)
Supplement: Supplementary file 1 [file Data_Sheet_1.docx]

Curli of Uropathogenic *Escherichia coli* Enhance Urinary Tract Colonization as a Fitness Factor

Víctor M. Luna-Pineda^1^, Leticia Moreno-Fierros^2^, Vicenta Cázares-Domínguez^1^, Damaris Ilhuicatzi-Alvarado^2^, Sara A. Ochoa^1^, Ariadnna Cruz-Córdova^1^, Pedro Valencia-Mayoral^3^, Alejandra Rodríguez-Leviz^3^, Juan Xicohtencatl-Cortes^1^

^1^ Laboratorio de Investigación en Bacteriología Intestinal, Hospital Infantil de México "Federico Gómez”, CDMX, México

^2^ Laboratorio de Inmunidad en Mucosas, Unidad de Biomedicina, Facultad de Estudios Superiores Iztacala, Universidad Nacional Autónoma de México, Tlalnepantla, Estado de México, México

^3^ Departamento de Patología, Hospital Infantil de México "Federico Gómez”, CDMX, México

**Supplementary Figures and Tables**

**
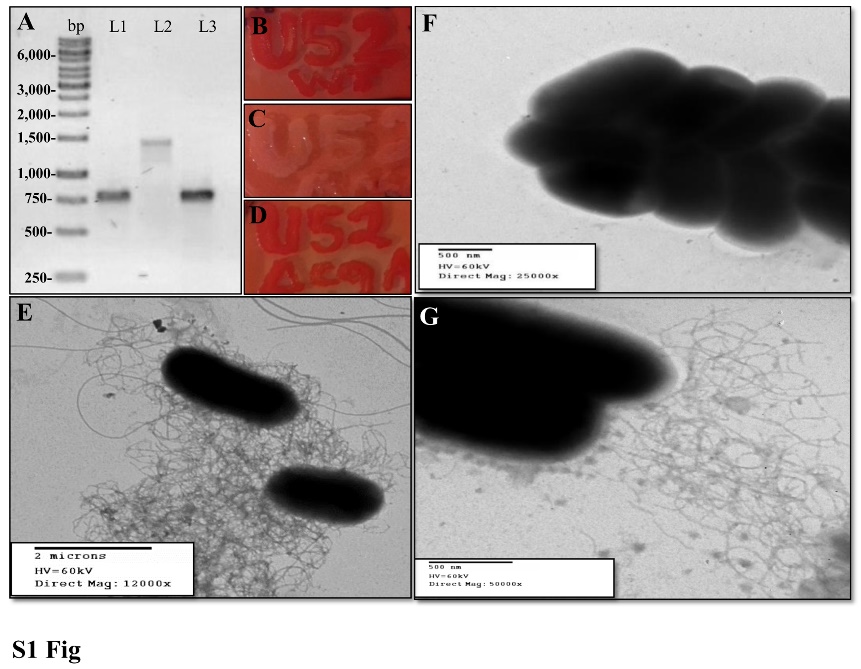
**

**Supplementary Figure 1. Genotypic and Phenotypic Evaluation of UPEC Strains.** (A) Verification of the *csgA* gene in UPEC strains visualized in a 0.7% agarose gel stained with ethidium bromide. Lane 1, wild-type strain (780 bp); lane 2, mutant strain (1400 bp); and lane 3, complemented strain (780 bp). (B) The wild-type strain showed red coloration, indicating the expression of curli. (C) The mutant strain lacked this phenotype due to the absence of curli expression. (D) The complemented strain showed a restored phenotype. MET micrographs show the presence of fimbriae in (E) the wild-type strain and (G) complemented strain. (F) The mutant strain showed the absence of characteristic curli structures. The gel image was edited, using clear delineation either with dividing lines and white space, while the original image is available in the follow link: [https://github.com/vlunapineda/Frontiers-in-Cell-and-Infect-Microb/blob/master/Supplementary%20Figure% 201.jpg](https://github.com/vlunapineda/Frontiers-in-Cell-and-Infect-Microb/blob/master/Supplementary%20Figure%25%20201.jpg)


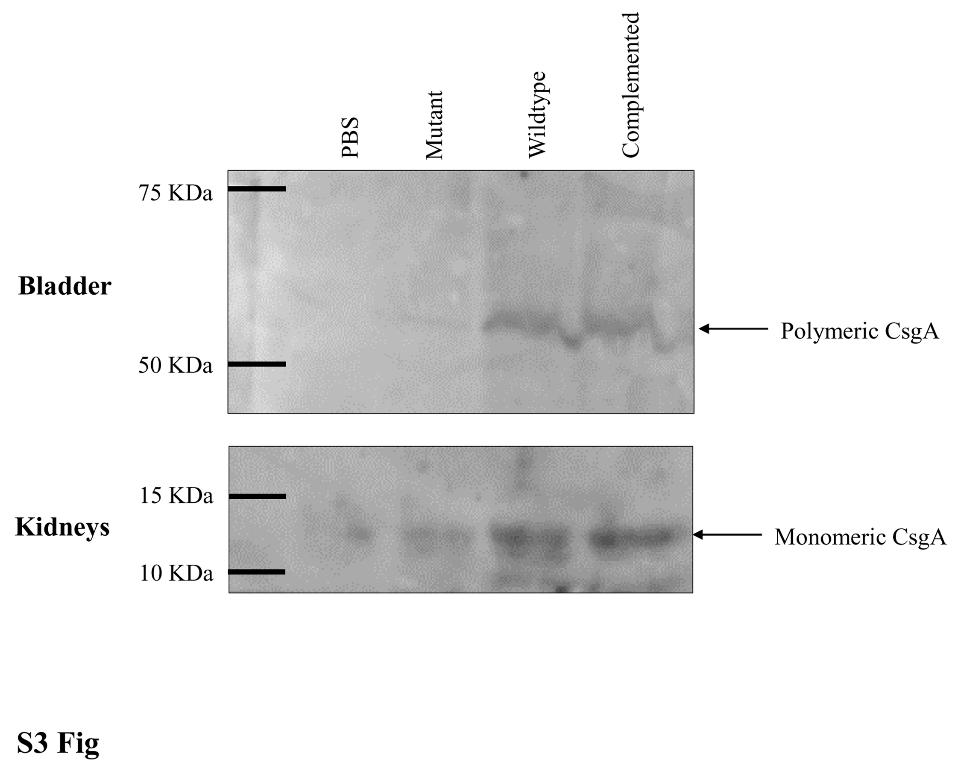


**Supplementary Figure 2. Curli is Expressed During UPEC Infection in both the Bladder and Kidney.** Western blot assays with the primary antibody anti-CsgA and the secondary antibody anti-IgG coupled with HRP. In the bladder, polymeric CsgA was identified in the wild-type and complemented strains at ~60 kDa. In the kidney, monomeric CsgA was also identified in the wild-type and complemented strains at ~12 kDa. The blot image was edited, using clear delineation either with dividing lines and white space, while the original image was available in the follow link: <https://github.com/vlunapineda/Frontiers-in-Cell-and-Infect-microb/blob/master/Supplementary%20Figure%202.Tif>

**
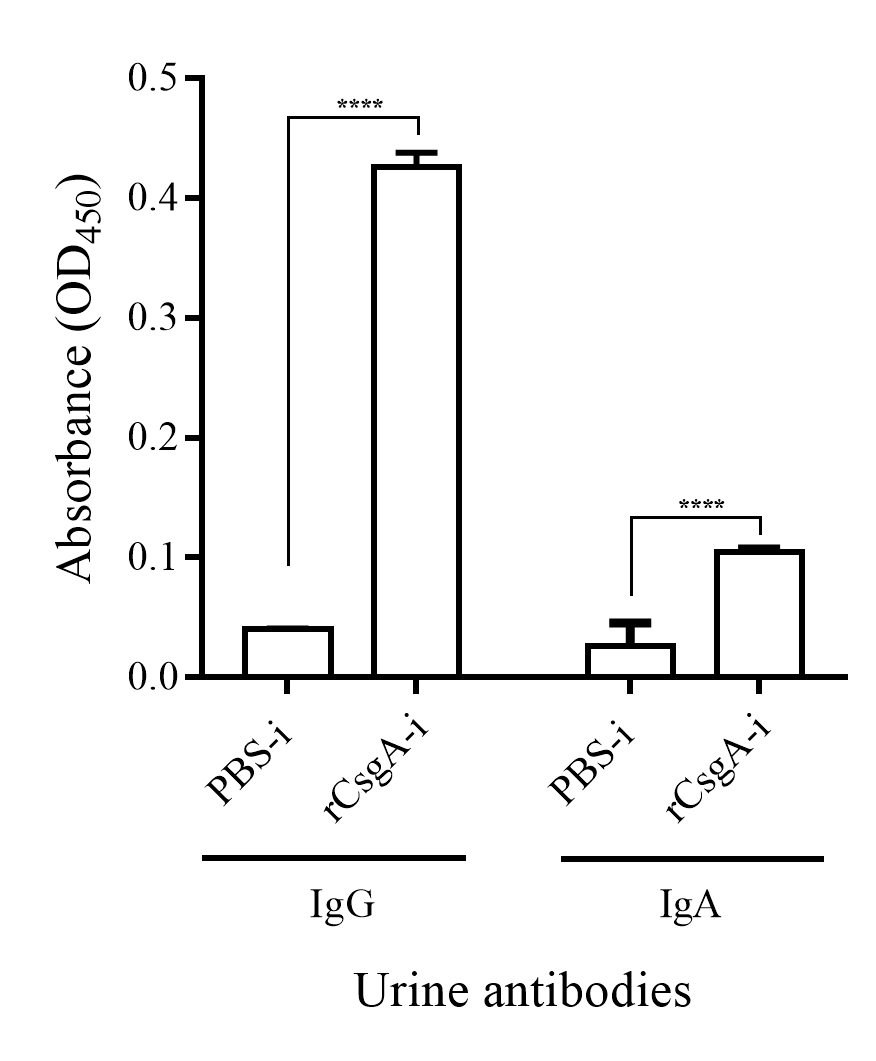
**

**Supplementary Figure 3. Identification of anti-rCsgA antibodies in urine.** C57BL/6 mice were immunized IN with the rCsgA protein and absorbance were determined by ELISA at 450 nm. The determination of antibodies against the rCsgA protein was performed with undiluted urine, using anti-IgG and anti-IgA coupled with HRP. IN immunization with PBS was used as negative control.


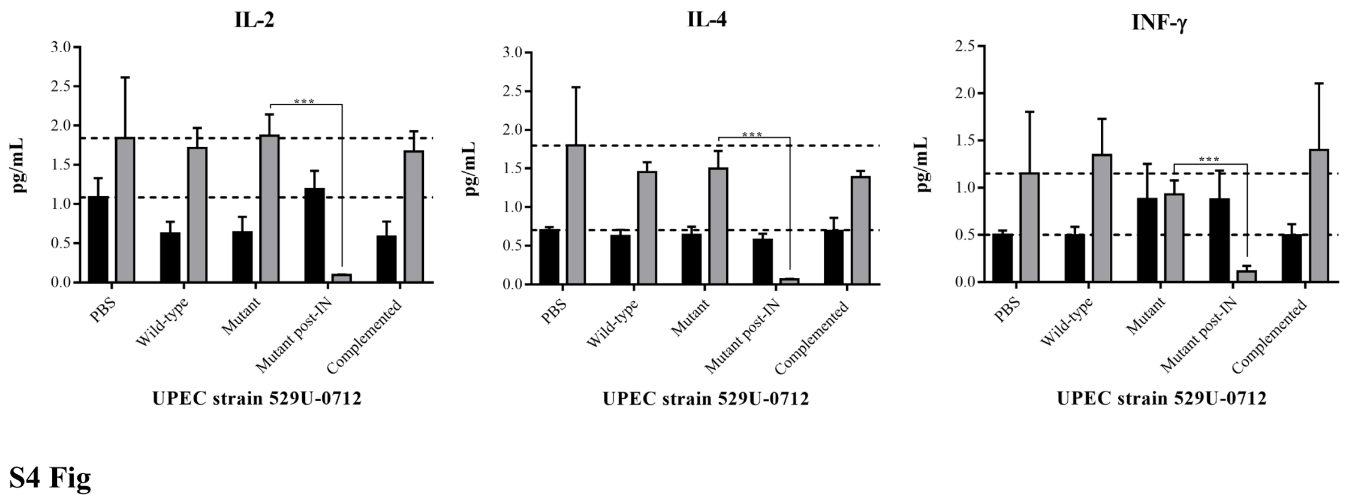


**Supplementary Figure 4. Quantification of IL-2, IL-4 and TNF-γ Release in the Mouse Bladder and Kidney.** Bladders (black bars) and kidneys (gray bars) were obtained, weighed, cut, and sonicated. The organ lysates were centrifuged, and the supernatants were used to quantify IL-2, IL-4 and TNF-γ. The UPEC clinical strain 529U-0712 was defined as the wild-type, the UPEC strain 529U-0712 *csgA*::Cm was defined as the mutant, and its complementation with the plasmid pJcsgG-C resulted in the complemented strain. Additionally, an IN immunization following infection with mutant strain was included and defined as mutant post-IN. Transurethral inoculation with PBS was used as a control in this experiment.

|  | Strain | Genes | | Expression condition | | | | |
| --- | --- | --- | --- | --- | --- | --- | --- | --- |
|  |  | ***csgA*** | ***bcsA*** | **Culture medium** | **Temperature** | **Time** | **Osmolality** | **pH** |
| 1 | 909U-0612 | + | - | *Brucella* agar | 37°C | 72hrs | 0.50% | 6 |
| 2 | 54U-0612 | + | + | YESCA agar | 37°C | 24hrs | 0.50% | 6 |
| 3 | 965U5-0412 | + | - | YESCA agar | 28°C and 37°C | 24 hrs | 0.50% | 6 |
| 4 | 117U1-0512 | + | + | YESCA agar | 28°C | 72hrs | 0.50% | 6 |
| 5 | 54U1-0512 | + | + | *Brucella* agar | 28°C and 37°C | 24 hrs | 0.50% | 6 |
| 6 | 807U1-0412 | + | - | *Brucella* agar | 37°C | 24 hrs | 0.50% | 6 |
| 7 | 310U5-0512 | + | + | YESCA agar | 28°C and 37°C | 24 hrs | 0.50% | 6 |
| 8 | 502U1-0412 | + | - | YESCA agar | 28°C and 37°C | 24 hrs | 0.50% | 6 |
| 9 | 494U2-0412 | + | + | YESCA agar | 28°C and 37°C | 24 hrs | 0.50% | 6 |
| 10 | 804U3-0412 | + | - | YESCA agar | 28°C and 37°C | 24 hrs | 0.50% | 6 |
| 11 | 173U5-0512 | + | - | *Brucella* agar | 37°C | 48 hrs | 0.50% | 6 |
| 12 | 657U-0612 | + | + | *Brucella* agar | 37°C | 48 hrs | 0.50% | 6 |
| 13 | 446U-0912 | + | - | YESCA agar | 37°C | 72 hrs | 0.50% | 6 |
| 14 | 249U-1012 | + | + | YESCA agar | 37°C | 48 hrs | 0.50% | 6 |
| 15 | 529U-0712 | + | + | YESCA agar | 28°C and 37°C | 24 hrs | 0.50% | 6 |
| 16 | 647U-0712 | + | + | YESCA agar | 28°C and 37°C | 24 hrs | 0.50% | 6 |
| 17 | 562U-0912 | + | + | YESCA agar | 37°C | 72 hrs | 0.50% | 6 |
| 18 | 877U | + | - | YESCA agar | 28°C and 37°C | 48 hrs | 0.50% | 6 |
| 19 | 179U-1012 | + | + | YESCA agar | 28°C and 37°C | 24 hrs | 0.50% | 6 |
| 20 | 992U-0912 | + | - | YESCA agar | 28°C and 37°C | 24 hrs | 0.50% | 6 |
| 21 | 513U-0912 | + | - | YESCA agar | 28°C and 37°C | 24 hrs | 0.50% | 6 |

**Supplementary Table 1. Expression Conditions of *csgA* Gene-Harboring UPEC Strains.**

**Supplemental Information table 2. Two-Way ANOVA Analysis of Cytokine Release by UPEC Strains.**

| Bladder | IL-10 | IL-17A | TNF | INF | IL-6 | IL-4 | IL-2 |
| --- | --- | --- | --- | --- | --- | --- | --- |
| PBS vs Wild-type | 0.1912 | 0.5289 | 0.8243 | 0.9846 | 0.9573 | 0.6604 | **0.0278** |
| PBS vs Mutant | 0.4703 | **<0.0001** | **0.0057** | 0.1105 | 0.6236 | 0.7124 | **0.0335** |
| PBS vs Mutant post-IN | **<0.0001** | **0.0068** | 0.5647 | 0.1138 | 0.9596 | 0.4656 | 0.6008 |
| PBS vs Complemented | 0.3717 | 0.1209 | 0.3151 | 0.9855 | 0.9257 | 0.9605 | **0.0172** |
| Wild-type vs Mutant | 0.5514 | **<0.0001** | **0.0102** | 0.1064 | 0.6618 | 0.9434 | 0.9359 |
| Wild-type vs Mutant post-IN | **<0.0001** | **0.0324** | 0.7228 | 0.1097 | 0.9977 | 0.7702 | **0.0076** |
| Wild-type vs Complemented | 0.6722 | 0.3481 | 0.432 | 0.9991 | 0.9683 | 0.6966 | 0.8394 |
| Mutant vs Mutant post-IN | **<0.0001** | **0.0032** | **0.0245** | 0.9875 | 0.6597 | 0.7166 | **0.0094** |
| Mutant vs Complemented | 0.8623 | **<0.0001** | 0.0645 | 0.1066 | 0.6907 | 0.7496 | 0.7772 |
| Mutant post-IN vs Complemented | **<0.0001** | 0.2122 | 0.6647 | 0.1099 | 0.966 | 0.4961 | **0.0045** |
| Kidney |  |  |  |  |  |  |  |
| PBS vs Wild-type | 0.3375 | 0.9287 | **<0.0001** | 0.4046 | 0.5949 | **0.046** | 0.5454 |
| PBS vs Mutant | **0.0002** | 0.895 | 0.4217 | 0.3477 | **<0.0001** | 0.0811 | 0.886 |
| PBS vs Mutant post-IN | **<0.0001** | 0.3762 | 0.0921 | **<0.0001** | 0.9627 | **<0.0001** | **<0.0001** |
| PBS vs Complemented | 0.135 | 0.9913 | **0.0003** | 0.2896 | 0.0519 | **0.0187** | 0.4077 |
| Wild-type vs Mutant | **0.0037** | 0.8248 | **0.0001** | 0.0806 | **<0.0001** | 0.7883 | 0.4552 |
| Wild-type vs Mutant post-IN | **<0.0001** | 0.4257 | **<0.0001** | **<0.0001** | 0.5631 | **<0.0001** | **<0.0001** |
| Wild-type vs Complemented | 0.5821 | 0.92 | 0.296 | 0.8187 | 0.1501 | 0.6968 | 0.8217 |
| Mutant vs Mutant post-IN | **<0.0001** | 0.3103 | **0.0152** | **0.0011** | **<0.0001** | **<0.0001** | **<0.0001** |
| Mutant vs Complemented | **0.0156** | 0.9036 | **0.0031** | **0.0498** | **0.0001** | 0.5113 | 0.3325 |
| Mutant post-IN vs Complemented | **<0.0001** | 0.3705 | **<0.0001** | **<0.0001** | **0.0469** | **<0.0001** | **<0.0001** |
